# Aqua­(2,2′-bipyridine)trifluorido­chromium(III) dihydrate

**DOI:** 10.1107/S1600536809031808

**Published:** 2009-08-19

**Authors:** Hai-Xing Liu

**Affiliations:** a Microscale Science Institute, Weifang University, Weifang 261061, People’s Republic of China, and Department of Chemistry and Chemical Engineering, Weifang University, Weifang 261061, People’s Republic of China

## Abstract

The title compound, [CrF_3_(C_10_H_8_N_2_)(H_2_O)]·2H_2_O, was prepared by the reaction of CrF_3_ and 2,2′-bipyridine under hydrous conditions. The metal centre is coordinated in a distorted octahedral mode by two N atoms from the organic ligand, three F atoms and one O atom of a water molecule. . The crystal packing is stabilized by O—H⋯O and O—H⋯F hydrogen-bonding contacts, which form a one-dimensional belt extending parallel to (100).

## Related literature

For anion structures, see: Kumar *et al.* (2007 ▶); Krishnan *et al.* (2007 ▶); Wu *et al.* (2007 ▶); Dong *et al.* (2005 ▶). For related structures, see: Timco *et al.* (2005 ▶); Larsen *et al.* (2003 ▶); Ochsenbein *et al.* (2008 ▶).
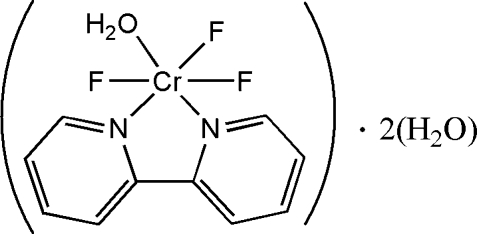


## Experimental

### 

#### Crystal data

[CrF_3_(C_10_H_8_N_2_)(H_2_O)]·2H_2_O
*M* *_r_* = 319.23
Monoclinic, 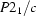

*a* = 9.0100 (18) Å
*b* = 7.4170 (15) Å
*c* = 20.759 (6) Å
β = 112.35 (3)°
*V* = 1283.1 (5) Å^3^
*Z* = 4
Mo *K*α radiation
μ = 0.93 mm^−1^
*T* = 293 K
0.24 × 0.18 × 0.17 mm

#### Data collection

Bruker SMART CCD area-detector diffractometer
Absorption correction: none
6478 measured reflections
2257 independent reflections
1916 reflections with *I* > 2σ(*I*)
*R*_int_ = 0.023

#### Refinement

*R*[*F* ^2^ > 2σ(*F* ^2^)] = 0.040
*wR*(*F* ^2^) = 0.129
*S* = 1.12
2257 reflections
175 parameters
3 restraints
H-atom parameters constrained
Δρ_max_ = 0.52 e Å^−3^
Δρ_min_ = −0.56 e Å^−3^

### 

Data collection: *SMART* (Bruker, 1997 ▶); cell refinement: *SAINT* (Bruker, 1997 ▶); data reduction: *SAINT*; program(s) used to solve structure: *SHELXS97* (Sheldrick, 2008 ▶); program(s) used to refine structure: *SHELXL97* (Sheldrick, 2008 ▶); molecular graphics: *SHELXTL* (Sheldrick, 2008 ▶); software used to prepare material for publication: *SHELXTL*.

## Supplementary Material

Crystal structure: contains datablocks global, I. DOI: 10.1107/S1600536809031808/br2114sup1.cif

Structure factors: contains datablocks I. DOI: 10.1107/S1600536809031808/br2114Isup2.hkl

Additional supplementary materials:  crystallographic information; 3D view; checkCIF report

## Figures and Tables

**Table 1:** Hydrogen-bond geometry (Å, °)

*D*—H⋯*A*	*D*—H	H⋯*A*	*D*⋯*A*	*D*—H⋯*A*
O1*W*—H2*W*1⋯F1	0.85	2.22	2.699 (3)	116
O1*W*—H2*W*1⋯F2^i^	0.85	2.02	2.567 (3)	121
O1*W*—H2*W*1⋯F2^i^	0.85	2.02	2.567 (3)	121
O1*W*—H1*W*1⋯F1^ii^	0.85	1.97	2.550 (3)	125
O2*W*—H1*W*2⋯F3^ii^	0.85	2.10	2.664 (4)	124
O2*W*—H2*W*2⋯O3*W*^iii^	0.85	2.33	2.730 (5)	110
O3*W*—H2*W*3⋯F2^iv^	0.80	1.98	2.767 (3)	171
O3*W*—H1*W*3⋯O3*W*^v^	0.84	2.18	2.748 (7)	125

Symmetry codes: (i) 
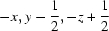
; (ii) 
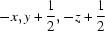
; (iii) 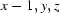; (iv) 
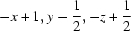
; (v) 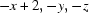.

**Fig. 1.**
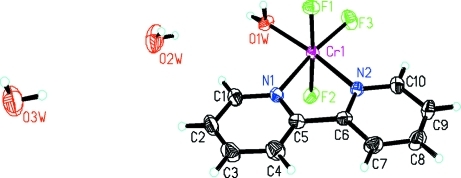
The molecular structure of the title compound with the atom-labeling scheme. Displacement ellipsoids are drawn at the 30% probability level.

**Fig. 2.**
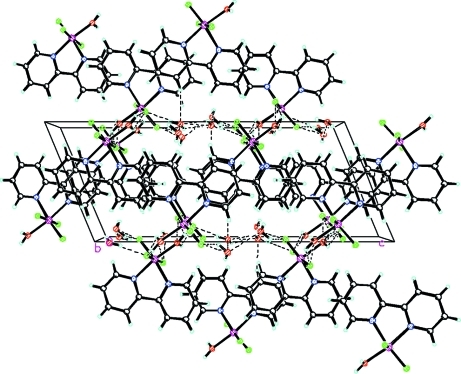
The packing view of the molecules of (I) along the crystallographic b direction.

## supplementary crystallographic information

### Comment

In recent, the aspect of anion attracts much research interesting in coordination chemistry, like *X*^-^, NO_3_^-^(Kumar *et al.*, 2007), BF_4_^-^, ClO_4_^-^(Krishnan *et al.*, 2007), SO_3_^2-^(Wu *et al.*, 2007). The anion components facilely either coordinate to metal atoms or fill the vacancy of Metal-organic frameworks, and intensively influence the supramolecular framework by hydrogen bonding and electrostatic interactions. But the study of F^-^ anion is still deficient. Because the HF strong acid easily attacks the glass surface and creates SiF_6_^2-^ in the synthetical progress. Here we describe the synthesis and structure of the title Cr compound coordinating with F atom.

The title structure (Fig. 1) was build up of one Cr atom, one 2,2,-bipyridine ligand, three coordination F atoms, one coordination water molecule and two free water molecules. Cr atom is coordinated with two N atoms from 2,2'-bipyridine ligand, three F atoms, one water molecule, presenting a distorted octahedron geometry. The mean Cr—N, Cr—O and Cr—F bond lengths are similar to the reported (Timco *et al.*, 2005, Larsen *et al.*, 2003 & Ochsenbein *et al.*, 2008). The torsion angles of C1—N1—Cr1—O1w, C10—N2—Cr1—F3 are 4.13 (2) and -4.25 (2)°,respectively.

The free water molecules link each other by intermolecular O—H···O hydrogen bonds. And F atoms contact with water molecules *via* intermolecular O—H···F hydrogen bonds (Table 2). The hydrogen-bonding interactions display as the one-dimensional belt linking the the crystal packing as shown in Fig. 2.

### Experimental

All commercially obtained reagent-grade chemicals were used without further purification. The novelty Cr(OH)_3_ was prepared by mixture CrCl_3_ 6H_2_O (5.33 g, 20 mmol) with NaOH (2.40 g, 60 mmol) in water solution. After filtered and washed with water, Cr(OH)_3_ was added to hydrofluoric acid (1.20 g, 60 mmol). The stirring did not stop until the solid dissolved completely. The CrF_3_ solution was obtained after increasing the pH value from 5 to 7. Ten drops of prepared CrF_3_ solution were added in the solution of 2,2'-bipyridine (0.48 g, 3 mmol) in water and methanol (3:1 *v*/*v*, 40 ml). The resulting solution was refluxed for 2 h and filtered. The brown prism crystals were collected, after cooling and filtering (yield 1.10 g).

### Refinement

H atoms were positioned geometrically and allowed to ride on their parent atoms, with C-H and O-H distances of 0.93–0.96 and 0.85 Å, respectively, and with *U*_iso_(H) = 1.2*U*_eq_ of the parent atoms.

### Crystal data

**Table:**

[CrF_3_(C_10_H_8_N_2_)(H_2_O)]·2H_2_O	*F*(000) = 652
*M**_r_* = 319.23	*D*_x_ = 1.653 Mg m^−^^3^
Monoclinic, *P*2_1_/*c*	Mo *K*α radiation, λ = 0.71073 Å
Hall symbol: -P 2ybc	Cell parameters from 1024 reflections
*a* = 9.0100 (18) Å	θ = 2.4–25.0°
*b* = 7.4170 (15) Å	µ = 0.93 mm^−^^1^
*c* = 20.759 (6) Å	*T* = 293 K
β = 112.35 (3)°	Prism, brown
*V* = 1283.1 (5) Å^3^	0.24 × 0.18 × 0.17 mm
*Z* = 4	

### Data collection

**Table:**

Bruker SMART CCD area-detector diffractometer	1916 reflections with *I* > 2σ(*I*)
Radiation source: fine-focus sealed tube	*R*_int_ = 0.023
graphite	θ_max_ = 25.0°, θ_min_ = 2.4°
φ and ω scans	*h* = −10→10
6478 measured reflections	*k* = −8→8
2257 independent reflections	*l* = −21→24

### Refinement

**Table:**

Refinement on *F*^2^	Primary atom site location: structure-invariant direct methods
Least-squares matrix: full	Secondary atom site location: difference Fourier map
*R*[*F*^2^ > 2σ(*F*^2^)] = 0.040	Hydrogen site location: inferred from neighbouring sites
*wR*(*F*^2^) = 0.129	H-atom parameters constrained
*S* = 1.12	*w* = 1/[σ^2^(*F*_o_^2^) + (0.0755*P*)^2^ + 0.6391*P*] where *P* = (*F*_o_^2^ + 2*F*_c_^2^)/3
2257 reflections	(Δ/σ)_max_ < 0.001
175 parameters	Δρ_max_ = 0.52 e Å^−^^3^
3 restraints	Δρ_min_ = −0.56 e Å^−^^3^

### Special details

**Table:**

Geometry. All e.s.d.'s (except the e.s.d. in the dihedral angle between two l.s. planes) are estimated using the full covariance matrix. The cell e.s.d.'s are taken into account individually in the estimation of e.s.d.'s in distances, angles and torsion angles; correlations between e.s.d.'s in cell parameters are only used when they are defined by crystal symmetry. An approximate (isotropic) treatment of cell e.s.d.'s is used for estimating e.s.d.'s involving l.s. planes.
Refinement. Refinement of *F*^2^ against ALL reflections. The weighted *R*-factor *wR* and goodness of fit *S* are based on *F*^2^, conventional *R*-factors *R* are based on *F*, with *F* set to zero for negative *F*^2^. The threshold expression of *F*^2^ > σ(*F*^2^) is used only for calculating *R*-factors(gt) *etc*. and is not relevant to the choice of reflections for refinement. *R*-factors based on *F*^2^ are statistically about twice as large as those based on *F*, and *R*- factors based on ALL data will be even larger.

### Fractional atomic coordinates and isotropic or equivalent isotropic displacement parameters (Å^2^)

**Table:**

	*x*	*y*	*z*	*U*_iso_*/*U*_eq_	
Cr1	0.15638 (5)	0.28271 (7)	0.32953 (2)	0.0283 (2)	
F1	0.2014 (2)	0.0442 (2)	0.31271 (9)	0.0393 (5)	
F2	0.1128 (2)	0.5267 (2)	0.34286 (9)	0.0436 (5)	
F3	−0.0083 (2)	0.2083 (3)	0.35635 (12)	0.0521 (6)	
N1	0.3615 (3)	0.3591 (4)	0.31451 (13)	0.0331 (6)	
N2	0.3252 (3)	0.2663 (3)	0.42877 (13)	0.0318 (6)	
C1	0.3689 (4)	0.4133 (5)	0.25417 (18)	0.0453 (8)	
H1A	0.2741	0.4299	0.2157	0.054*	
C2	0.5145 (5)	0.4453 (5)	0.2477 (2)	0.0540 (10)	
H2A	0.5168	0.4829	0.2054	0.065*	
C3	0.6529 (5)	0.4212 (6)	0.3035 (2)	0.0579 (10)	
H3A	0.7513	0.4406	0.2997	0.069*	
C4	0.6469 (4)	0.3678 (6)	0.3661 (2)	0.0533 (10)	
H4A	0.7410	0.3513	0.4050	0.064*	
C5	0.4997 (4)	0.3393 (4)	0.37029 (17)	0.0358 (7)	
C6	0.4791 (4)	0.2890 (4)	0.43569 (17)	0.0353 (7)	
C7	0.6048 (5)	0.2693 (5)	0.4996 (2)	0.0506 (10)	
H7A	0.7105	0.2842	0.5037	0.061*	
C8	0.5698 (6)	0.2272 (6)	0.5569 (2)	0.0613 (12)	
H8A	0.6521	0.2127	0.6003	0.074*	
C9	0.4128 (5)	0.2068 (5)	0.54970 (18)	0.0559 (11)	
H9A	0.3875	0.1802	0.5881	0.067*	
C10	0.2933 (5)	0.2264 (5)	0.48461 (18)	0.0432 (8)	
H10A	0.1870	0.2113	0.4796	0.052*	
O1W	0.0184 (3)	0.3056 (3)	0.22962 (11)	0.0363 (5)	
H1W1	−0.0536	0.3443	0.1923	0.044*	
H2W1	0.0476	0.2023	0.2211	0.044*	
O2W	0.0891 (5)	0.4622 (5)	0.06932 (16)	0.0883 (11)	
H1W2	0.0245	0.4742	0.0901	0.106*	
H2W2	0.1487	0.3719	0.0871	0.106*	
O3W	0.9992 (4)	0.1082 (5)	0.05359 (17)	0.0790 (10)	
H2W3	0.9750	0.0764	0.0850	0.095*	
H1W3	1.0333	0.0066	0.0477	0.095*	

### Atomic displacement parameters (Å^2^)

**Table:**

	*U*^11^	*U*^22^	*U*^33^	*U*^12^	*U*^13^	*U*^23^
Cr1	0.0252 (3)	0.0273 (3)	0.0288 (3)	0.00016 (18)	0.0059 (2)	0.00170 (18)
F1	0.0344 (10)	0.0296 (10)	0.0462 (10)	0.0000 (8)	0.0068 (8)	−0.0007 (8)
F2	0.0530 (12)	0.0329 (10)	0.0371 (10)	0.0091 (9)	0.0084 (9)	−0.0017 (8)
F3	0.0343 (11)	0.0647 (15)	0.0608 (13)	−0.0003 (10)	0.0221 (10)	0.0137 (10)
N1	0.0326 (14)	0.0322 (14)	0.0334 (14)	−0.0042 (11)	0.0113 (11)	0.0016 (11)
N2	0.0322 (14)	0.0298 (14)	0.0288 (14)	−0.0015 (11)	0.0063 (11)	0.0028 (10)
C1	0.048 (2)	0.046 (2)	0.0418 (19)	−0.0066 (17)	0.0172 (16)	0.0051 (15)
C2	0.068 (3)	0.050 (2)	0.059 (2)	−0.0085 (19)	0.042 (2)	0.0035 (18)
C3	0.043 (2)	0.066 (3)	0.072 (3)	−0.0106 (19)	0.030 (2)	−0.001 (2)
C4	0.0329 (18)	0.063 (2)	0.062 (2)	−0.0048 (18)	0.0159 (17)	0.000 (2)
C5	0.0315 (16)	0.0313 (16)	0.0425 (18)	−0.0038 (13)	0.0116 (14)	−0.0021 (14)
C6	0.0303 (17)	0.0296 (17)	0.0384 (18)	−0.0028 (13)	0.0046 (14)	−0.0014 (13)
C7	0.0354 (19)	0.052 (2)	0.046 (2)	−0.0042 (16)	−0.0048 (17)	0.0025 (16)
C8	0.062 (3)	0.062 (3)	0.037 (2)	−0.003 (2)	−0.0079 (19)	0.0087 (17)
C9	0.072 (3)	0.057 (3)	0.0294 (19)	−0.006 (2)	0.0090 (18)	0.0086 (16)
C10	0.045 (2)	0.045 (2)	0.0367 (19)	−0.0040 (16)	0.0128 (16)	0.0048 (15)
O1W	0.0351 (12)	0.0310 (11)	0.0305 (11)	0.0072 (9)	−0.0013 (9)	−0.0002 (9)
O2W	0.124 (3)	0.079 (2)	0.081 (2)	0.037 (2)	0.060 (2)	0.0043 (18)
O3W	0.096 (2)	0.083 (2)	0.079 (2)	−0.002 (2)	0.0559 (19)	0.0067 (19)

### Geometric parameters (Å, °)

**Table:**

Cr1—F3	1.856 (2)	C4—H4A	0.9300
Cr1—F1	1.8769 (18)	C5—C6	1.486 (5)
Cr1—F2	1.8942 (19)	C6—C7	1.386 (5)
Cr1—O1W	1.979 (2)	C7—C8	1.379 (6)
Cr1—N2	2.047 (3)	C7—H7A	0.9300
Cr1—N1	2.067 (3)	C8—C9	1.373 (6)
N1—C1	1.341 (4)	C8—H8A	0.9300
N1—C5	1.348 (4)	C9—C10	1.378 (5)
N2—C10	1.329 (4)	C9—H9A	0.9300
N2—C6	1.350 (4)	C10—H10A	0.9300
C1—C2	1.388 (5)	O1W—H1W1	0.8498
C1—H1A	0.9300	O1W—H2W1	0.8500
C2—C3	1.353 (6)	O2W—H1W2	0.8500
C2—H2A	0.9300	O2W—H2W2	0.8500
C3—C4	1.378 (6)	O3W—H2W3	0.7978
C3—H3A	0.9300	O3W—H1W3	0.840 (10)
C4—C5	1.378 (5)		
			
F3—Cr1—F1	91.69 (9)	C2—C3—H3A	120.3
F3—Cr1—F2	90.37 (10)	C4—C3—H3A	120.3
F1—Cr1—F2	177.37 (8)	C3—C4—C5	119.1 (4)
F3—Cr1—O1W	94.86 (10)	C3—C4—H4A	120.4
F1—Cr1—O1W	88.84 (8)	C5—C4—H4A	120.4
F2—Cr1—O1W	89.35 (8)	N1—C5—C4	121.7 (3)
F3—Cr1—N2	93.05 (10)	N1—C5—C6	114.7 (3)
F1—Cr1—N2	90.05 (9)	C4—C5—C6	123.6 (3)
F2—Cr1—N2	91.48 (9)	N2—C6—C7	121.4 (3)
O1W—Cr1—N2	172.04 (10)	N2—C6—C5	114.5 (3)
F3—Cr1—N1	171.69 (10)	C7—C6—C5	124.1 (3)
F1—Cr1—N1	87.80 (9)	C8—C7—C6	118.6 (4)
F2—Cr1—N1	90.39 (10)	C8—C7—H7A	120.7
O1W—Cr1—N1	93.42 (10)	C6—C7—H7A	120.7
N2—Cr1—N1	78.66 (11)	C9—C8—C7	119.7 (4)
C1—N1—C5	118.5 (3)	C9—C8—H8A	120.2
C1—N1—Cr1	126.0 (2)	C7—C8—H8A	120.2
C5—N1—Cr1	115.3 (2)	C8—C9—C10	118.9 (4)
C10—N2—C6	119.3 (3)	C8—C9—H9A	120.5
C10—N2—Cr1	124.5 (2)	C10—C9—H9A	120.5
C6—N2—Cr1	116.1 (2)	N2—C10—C9	122.1 (4)
N1—C1—C2	121.7 (3)	N2—C10—H10A	119.0
N1—C1—H1A	119.1	C9—C10—H10A	119.0
C2—C1—H1A	119.1	Cr1—O1W—H1W1	160.5
C3—C2—C1	119.5 (3)	Cr1—O1W—H2W1	91.2
C3—C2—H2A	120.3	H1W1—O1W—H2W1	107.7
C1—C2—H2A	120.3	H1W2—O2W—H2W2	107.7
C2—C3—C4	119.4 (4)	H2W3—O3W—H1W3	94.7

### Hydrogen-bond geometry (Å, °)

**Table:**

*D*—H···*A*	*D*—H	H···*A*	*D*···*A*	*D*—H···*A*
O1W—H2W1···F1	0.85	2.22	2.699 (3)	116
O1W—H2W1···F2^i^	0.85	2.02	2.567 (3)	121
O1W—H2W1···F2^i^	0.85	2.02	2.567 (3)	121
O1W—H1W1···F1^ii^	0.85	1.97	2.550 (3)	125
O2W—H1W2···F3^ii^	0.85	2.10	2.664 (4)	124
O2W—H2W2···O3W^iii^	0.85	2.33	2.730 (5)	110
O3W—H2W3···F2^iv^	0.80	1.98	2.767 (3)	171
O3W—H2W3···F3^iv^	0.80	2.96	3.490 (4)	126
O3W—H1W3···O3W^v^	0.84	2.18	2.748	125

Symmetry codes:  (i) −*x*, *y*−1/2, −*z*+1/2; (ii) −*x*, *y*+1/2, −*z*+1/2; (iii) *x*−1, *y*, *z*; (iv) −*x*+1, *y*−1/2, −*z*+1/2; (v) −*x*+2, −*y*, −*z*.
